# Virtual inverted classroom to replace in-person radiology lectures at the time of the COVID-19 pandemic - a prospective evaluation and historic comparison

**DOI:** 10.1186/s12909-021-03061-4

**Published:** 2021-12-11

**Authors:** Ulf Teichgräber, Birger Mensel, Tobias Franiel, Aimée Herzog, Chie-Hee Cho-Nöth, Hans-Joachim Mentzel, Maja Ingwersen, René Aschenbach

**Affiliations:** 1 Office of the Dean, Faculty of Medical Education, Friedrich Schiller University, Jena University Hospital, Jena, Germany; 2 Department of Radiology, Friedrich Schiller University, Jena University Hospital, Jena, Germany; 3 Department of Radiology, Zentralklinikum Bad Berka, Bad Berka, Germany

**Keywords:** Cohort study, COVID-19, Distance education and online learning, Inverted classroom, Radiology, Survey, Virtual classroom

## Abstract

**Background:**

In the time of the coronavirus disease 2019 (COVID-19) pandemic, in-person lectures had to be shifted to online learning. This study aimed to evaluate students’ and lecturers’ perception and effectiveness of a virtual inverted classroom (VIC) concept on clinical radiology in comparison to a historic control.

**Methods:**

In the winter semester 2020/21, 136 fourth year medical students who completed the clinical radiology VIC during the pandemic, were included in the single centre, prospective study. Results were compared with a historic control that had finished the physical inverted classroom (PIC) in the immediately preceding year. The VIC consisted of an initial phase of self-determined preparation with learning videos and a second interactive phase of clinical case studies alternating between the virtual lecture hall and virtual buzz groups. At the end of the lecture series, students rated the lecture on a scale of 1 (most positive assessment) to 6 (most negative assessment) through an online survey platform. Additionally, they reported their impressions in free-form text. Lecturers were invited to comment on the VIC in a group interview. Main outcomes were final grades and student perception of the VIC.

**Results:**

Students’ general impression of VIC was lower than that of PIC (median value of 3 [IQR 4, 2] and 1 [IQR 0, 0], p < 0.001), respectively, p < 0.001). The highest rating was achieved concerning use of the audience response system (median 1 [IQR 1, 0]), and the lowest concerning the buzz groups (median 4 [IQR 5, 3]). Students stated that they would have appreciated more details on reading images, greater focus on plenary case studies, and provision of exam related scripts. Lecturers would have liked better preparation by students, more activity of students, and stronger assistance for group support. Exam grades after VIC were better than after PIC (median 1 [IQR 2, 1] and 2 [IQR 2,1], respectively, p < 0.001).

**Conclusions:**

Students’ overall perception of VIC was satisfactory, although worse than PIC. Final grades improved compared to PIC. Provided an adapted buzz group size and support, VIC may serve as complement in medical education once the pandemic is over.

## Background

The coronavirus disease 2019 (COVID-19) pandemic considerably affected medical education. Social distancing was required and thus, face-to face learning had to be replaced from one day to the next by remote learning using online platforms, social media, and virtual classrooms [1]. Whether didactic approaches, developed out of necessity, may complement conventional concepts of medical education beyond the pandemic, remains unclear.

The year before the outbreak of the COVID-19 pandemic, we had implemented the didactic concept of inverted classroom in clinical radiology. At that time, we intended to improve the traditional teacher-centred lecture that is mainly based on transfer of factual knowledge. For this purpose, we had established a heutagogical didactic approach by combining self-determined multimedia-based [2] and subsequent cooperative elements [3, 4]. Heutagogy designates a self-determined and self-directed learning approach that promotes autonomy of students. Active participation and cooperation of the students was demanded during buzz group discussions and use of an audience response system (ARS). For buzz group discussions, participants of the lecture had been divided into small groups to briefly discuss key feature questions. Finally, students had assessed the didactic quality and learning success as better than students who had undergone the traditional radiology lecture in previous years. Lecturers had found an increased gain in professional skills among their students [5]. However, when the pandemic spread to Germany, we felt obliged to convert the physical inverted classroom (PIC) with its in-person interactive and collaborative elements into a virtual inverted classroom (VIC). An earlier study found that distance teaching increased flexibility and saved travel time [6]. The approach was efficacious regarding learning success [7, 8]. However, effectiveness depended on communication between teachers and students [9]. Moreover, virtual teaching is supposed to facilitate inter-centre didactic collaboration [10].

This study aimed to evaluate students’ and lecturers’ perception and effectiveness of remote medical education in clinical radiology using the approach of VIC during the COVID-19 pandemic. Lessons learned from the enforced experience with VIC may provide the opportunity to innovate and expand the didactic spectrum of medical curricula, regardless of further viral outbreaks.

## Methods

### Study design

**Fig. 1:**
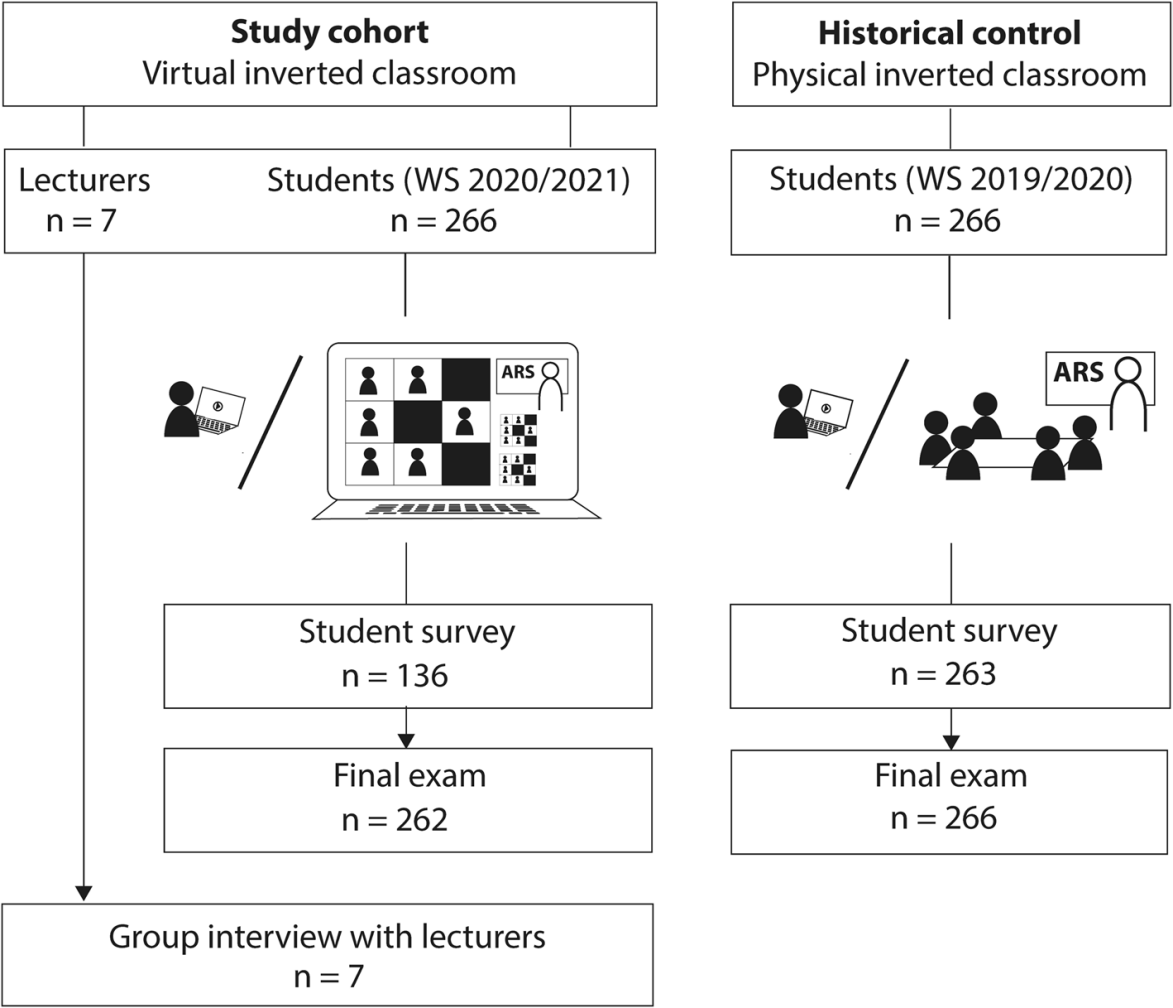
Flow chart of the comparison between virtual inverted classroom during the COVID-19 pandemic and the historical control of physical inverted classroom during the immediately preceding year. ARS: audience response system, WS: winter semester

During the COVID-19 pandemic we implemented the approach of VIC in clinical radiology at our university hospital. We included fourth year medical students who underwent the winter semester 2020/2021 radiology lecture series, and seven lecturers who covered six radiological topics in our prospective, observational study. At the end of the semester, before the final exam, we asked students to voluntary participate in an online survey on their impressions about the VIC. Student survey results and exam grades were then compared to those of a historical control group that had completed the PIC lecture in the winter semester 2019/2020 [5]. Lecturers were senior radiologists with a minimum of 5 years teaching experience after board exam. They were the same persons who had given the historical control of PIC. Upon completion of the lecture series, they were asked for a group interview on VIC. We anonymized all survey data from the study cohort and the historical control. The local ethics committee declared a waiver of obligation of approval. The study design is specified in Fig. 1.

### The inverted classroom

The concept of VIC arose from the necessity to avoid personal contacts due to the COVID-19 pandemic. Therefore, the previous approach of PIC, that had been described earlier [5] has been adapted accordingly.

The approach of inverted classroom is based on a heutagogic teaching model of blended learning [4, 11–13]. Initially, students are expected to prepare independently by online videos and additional self-chosen informational resources [2]. Thereafter, they needed to actively participate the classroom that was virtual in this study and physical for the historical control group. In any case, lecturers were charged with the task to define and communicate intended learning outcomes [14] and to prepare learning videos on their area of expertise accordingly. The videos provided asynchronous knowledge transfer to take students to the competence level of understanding according to Bloom’s taxonomy [15], which means to enable students to identify and describe radiological image content. Videos should have been no longer than 8 to 12 min and total preparatory time was recommended to last at least 45 min.

Thereafter, with VIC, the interactive classroom was held by means of an online conference meeting using Zoom (Zoom Video Communications, San Jose, California) that included a synchronous virtual plenary room with polling and chat applications, and virtual breakout rooms for asynchronous buzz group discussions. The aim of classroom activities was to take students to the next higher competence levels of application and analysis [15] that is in our case to interpret and assess radiological images, and to communicate radiological findings. Overall, we offered six virtual radiology classroom sessions of 30 min each covering a total of six topics. The virtual attendance phase of the lecture was inspired by the so-called sandwich-design of interactive lectures [16]. At the start of the classroom, students were requested to download radiological images that were intended to be object of plenary multiple-choice key-feature questions (KFQ). After a short description of a clinical case, lecturer raised a KFQ that needed to be discussed simultaneously within separate virtual breakout rooms in buzz groups consisting of 10 randomly selected students each. Discussions took about 5 min each. During the buzz group discussion, lecturer and the teaching assistant could virtually enter breakout rooms to professionally support buzz groups. Breakout room discussion was followed by voting on the KFQ by each single student by means of an online ARS. Anonymized responses were then debated plenary in virtual interaction with the lecturer. Each lecture comprises three to five above outlined units of student activation and collaboration in buzz groups and corresponding compact interaction with the lecturer in plenary. Administrative-technical personnel continuously supported coordination of virtual rooms and ARS.

In contrast, with PIC, students and lecturers had attended the classroom in person. Beforehand, the approach of PIC had been presented to students by means of an online podcast. Buzz groups had been formed of two to three students sitting next to each other in the lecture hall, equipped with a single televoting “clicker” per group for the ARS. Analysis of votes had been displayed on a screen in the lecture hall [5]. Either way, at the end of the lecture, as an exit point, lecturers needed to summarize the results of the clinical case study in plenary.

### Study outcomes

Outcomes of equal weight were student rating of the entire lecture series comprising eleven statements to be evaluated and assessed with a degree of agreement on a scale of 1 (fully agree) to 6 (totally disagree) and students’ final grades, rated with marks from 1 (excellent) to 5 (inadequate). Ratings and grading were compared to the historic control of PIC. The following statements were assessed: content of lectures was aligned across the six lecture topics (1), learning objectives were stated clearly (2), didactic quality was high (3), lecturers were engaged (4), knowledge was gained (5), lecture subject was addressed (6), learning video was useful (7), ARS was useful (8), buzz group was useful (9), general impression was positive (10), and continuation of the teaching concept was desirable. The survey was conducted online (Lime survey: an open-source survey tool; LimeSurvey GmbH, Hamburg, Germany) with the VIC and had been carried out using a paper questionnaire with PIC. In addition, we evaluated students’ impressions that they had reported in free-form text, and students rating of every single topic of the lecture series using the valuation method described above. Students assessed the individual lecture topics according to the following statements: learning video was useful (1), ARS was useful (2), buzz group was useful (3), and clinical cases were useful (4). Finally, we evaluated lecturers’ assessment of different aspects of the VIC during a group interview.

Radiology examination did not differ between the PIC and VIC groups regarding the level of difficulty.

### Statistical analysis

Categorical variables are given as counts and percentages and continuous variables as means and standard deviations (SD). Medians and interquartile ranges (IQR) are provided for ordinal categorical variables. Comparison of ratings to the historical control (PIC) and exam grades were conducted using the Mann–Whitney U test. Nemenyi’s procedure was run for pairwise comparison of ratings concerning individual lectures of the VIC. Bonferroni correction was applied to set p-value cut-offs of 2-sided p at < 0.005 for the historic comparison and at < 0.013 for the pairwise comparison of VIC lecture topics. Analysis was performed using XLSTAT (Version 2015.6.01.24026, Addinsoft, Paris, France).

## Results

**Fig. 2:**
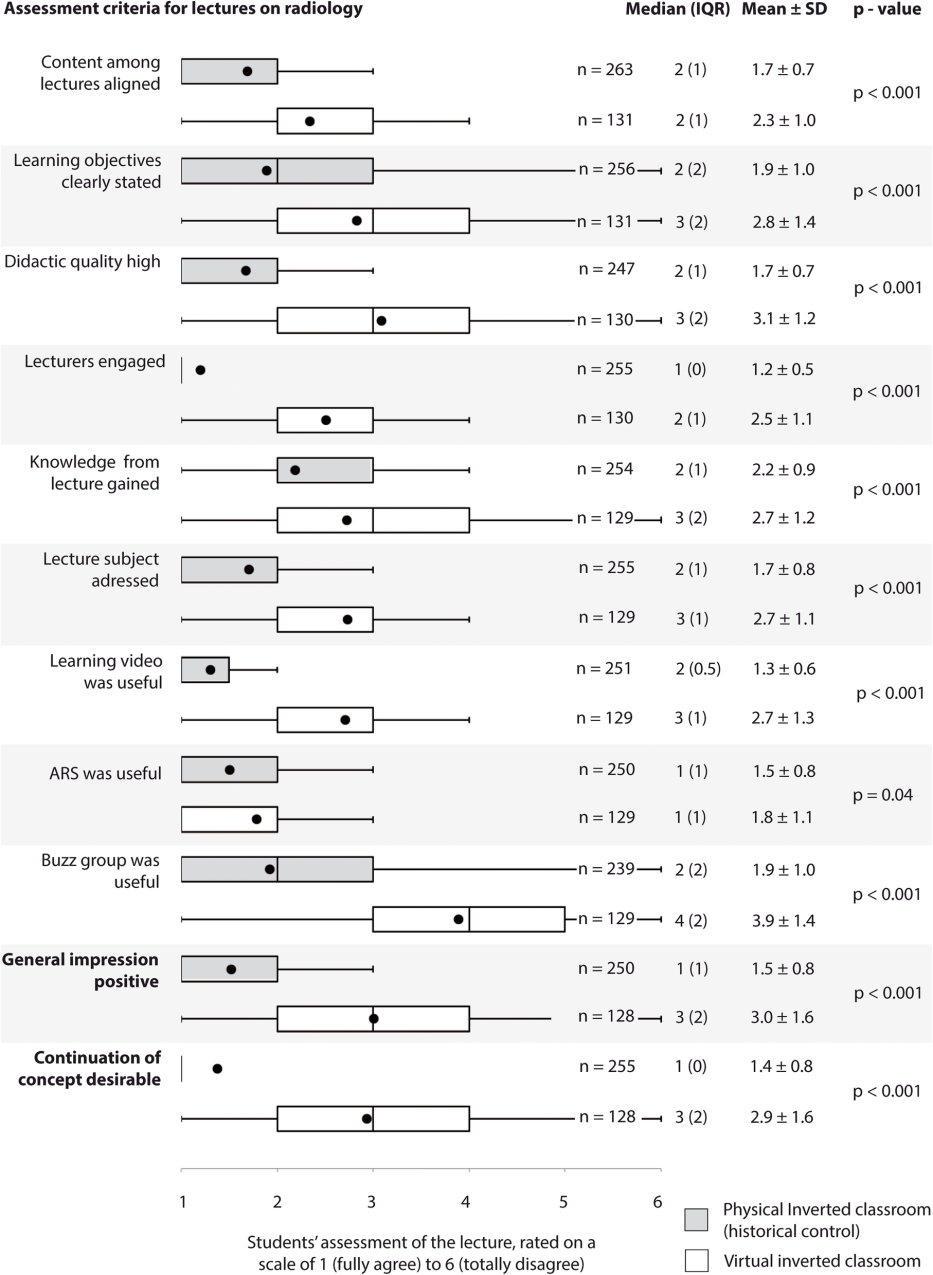
Comparison of students’ assessment of the concept of virtual inverted classroom with a historical control that assessed the concept of physical inverted classroom in the year preceding the pandemic. Box plots indicate median and interquartile range. Whiskers end with the lowest and highest data point within 1.5 × interquartile range. Means are represented as dots. P-value cut-off is set at 0.005 to correct for multiple testing. ARS: audience response system, IQR: interquartile range

A total of 136 of 266 (51.1%) students (24.5 ± 3.3 years old, 90 female) participated in the voluntary end-of-semester student survey (response rate of the historic control: 263 of 266 students). Students rated their general impression of the clinical radiology VIC with a median value of 3 (IQR 4, 2) on a scale of 1 (most positive assessment) to 6 (most negative assessment), which was significantly worse compared to the historic control, who had attended the PIC (median of 1 [IQR 0, 0], p < 0.001). Except for utility of ARS, all individual criteria including alignment of content, stating of learning objectives, didactic quality, engagement of lecturers, knowledge gained, learning video, and buzz group were rated lower with VIC than with PIC. Students gave their poorest rating to the buzz group (median of 4 [IQR 5, 3]) and their best to the ARS (median 1 [IQR 1, 0]) (Fig. 2).

**Fig. 3:**
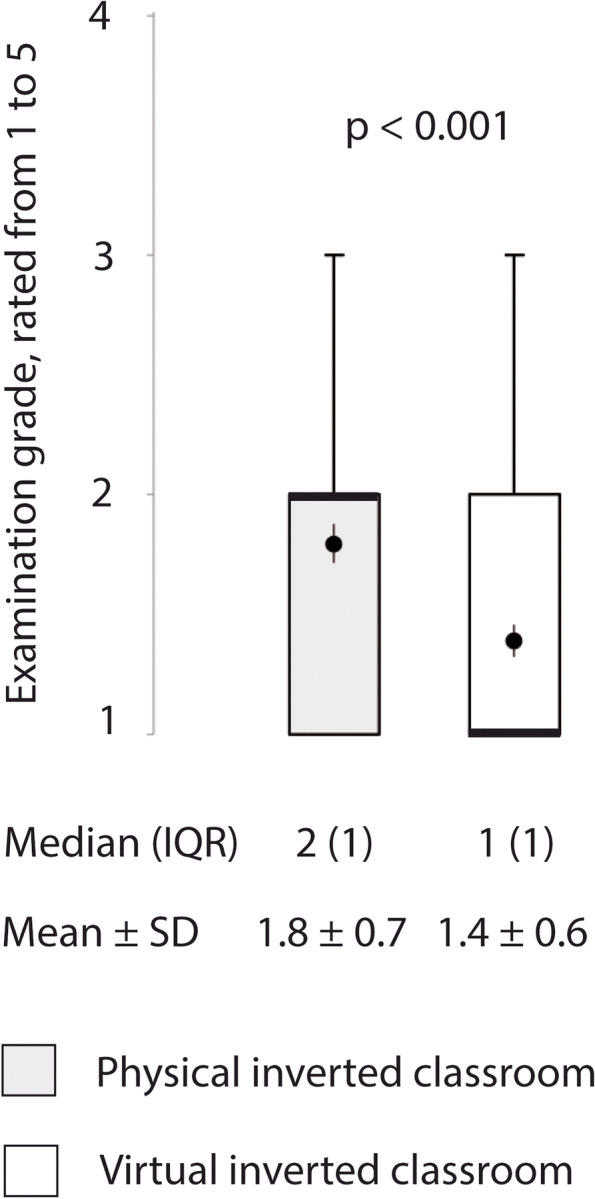
Students’ radiology examination grades from 1 (excellent) to 5 (inadequate) after lectures following the concept of virtual inverted classroom compared to a historical control following the concept of physical inverted classroom. Box plots indicate median and interquartile range. Whiskers end with the lowest and highest data point within 1.5 × interquartile range. Means with their 95% CI intervals are represented as dots

Average radiology examination grades of students following the VIC were better than those achieved with the PIC (median 1 [IQR 2, 1] and 2 [IQR 2,1], respectively), (Fig. 3).

Except for utility of the buzz group, students` assessment differed among individual lectures of the series. Concerning the learning video, students rated the lecture on gynaecological radiology poorer (median 3 [IQR 4, 3]) than each of the other lectures. Regarding ARS, a significant difference only occurred between gynaecological and gastroenterological radiology (median 2 [IQR 4, 2] and 1 [IQR 2, 1], respectively). Most pairwise differences across topics were seen on the issue of plenary clinical case studies, with the greatest difference between gynaecological and gastroenterological radiology (difference 1.3 [95%CI: 1.1 to 1.6], p < 0.001), (Table 1).

**Table 1:** Students’ Assessment of Individual Lectures of the Radiology Lecture Series Following the 2003Concept of Virtual Inverted Classroom

	**Number of replies**	**Median (IQR)**	**Mean ± SD**	**Pairwise** **comparison**
**Learning video was useful**				
Thorax	96	2 (1)	2.4 ± 1.0	p < 0.001^a^
Urology/MRT	92	2 (1.25)	2.3 ± 1.0	p < 0.001^a^
Gynaecology/Breast^f^	89	3 (1)	3.2 ± 1.2	p < 0.001^b^
Angiology/Intervention	94	2 (1)	2.4 ± 1.0	p < 0.001^a^
Gastroenterology	96	2 (1)	1.9 ± 1.0	p < 0.001^a^
Paediatrics	94	2 (1)	2.3 ± 1.1	p < 0.001^a^
**ARS was useful**				
Thorax	79	2 (2)	2.2 ± 1.2	ns
Urology/MRT	75	2 (2)	2.3 ± 1.3	ns
Gynaecology/Breast	72	2 (2)	2.7 ± 1.3	p < 0.001^c^
Angiology/Intervention	76	2 (2)	2.2 ± 1.2	ns
Gastroenterology	87	1 (1)	1.9 ± 1.3	p < 0.001^a^
Paediatrics	83	2 (2)	2.3 ± 1.3	ns
**Buzz group was useful**				
Thorax	83	4 (2)	4.0 ± 1.5	ns
Urology/MRT	78	4 (2)	4.0 ± 1.5	ns
Gynaecology/Breast	79	4 (2)	4.2 ± 1.4	ns
Angiology/Intervention	79	4 (2)	3.9 ± 1.5	ns
Gastroenterology	82	4 (3)	3.8 ± 1.6	ns
Paediatrics	80	4 (2)	3.9 ± 1.5	ns
**Plenary clinical case studies** **were useful**				
Thorax	90	2 (1)	2.4 ± 1.0	p = 0.004^a^ p = 0.001^c^
Urology/MRT	81	3 (1)	2.5 ± 1.0	p < 0.001^c^
Gynaecology/Breast	83	3 (2)	3.1 ± 1.1	p = 0.004^d^ p = 0.003^e^ p < 0.001^c^
Angiology/Intervention	85	2 (1)	2.4 ± 0.9	p = 0.003^a^ p = 0.001^c^
Gastroenterology	93	1 (1)	1.7 ± 1.1	p = 0.001^d, e^ p < 0.001^a^
Paediatrics	90	2 (1)	2.3 ± 1.0	p < 0.001^a^

Lectures were rated at a scale of 1 (fully agree) to 6 (totally disagree). P-value cut-off is set at 0.013 to correct for multiple testing

^a^ Concerning difference to gynaecology lecture

^b^ Concerning difference to all other lectures

^c^ Concerning difference to gastroenterology lecture

^d^ Concerning difference to thorax lecture

^e^ Concerning difference to angiology lecture

^f^ Video exceeded recommended length more than threefold

ARS: audience response system, ns: no significant difference to other lectures

In reply to the survey, students stated that they appreciated to independently prepare with learning videos, however expressed additional requests. These included the requirement to clearly indicate pathological structures on images, to provide healthy comparison images, and to give the opportunity to download high-resolution images. Students welcomed the possibility to ask questions during the VIC, but in addition would have liked more basic rather than specialist knowledge to be imparted. They encouraged lecturers to correct students’ misdiagnosis in plenary, that resulted from preceding discussions within the buzz groups. With VIC, more emphasis should be put on plenary than on breakout room discussions. Students considered the well-defined objectives that were set out at the start of lectures as positive. However, in addition they had wished to be provided with scrips, take-home-messages, references for further information, and an objectives catalogue that might have supported exam preparation (Table 2).

**Table 2:** Student Survey on the Approach of Virtual Inverted Classroom in Clinical Radiology

**Asynchronous learning**	**Synchronous learning**	**General**
**Positive aspects**		
•Learning videos can be viewed repeatedly	•Well-defined objectives at the start of lectures	•Large number of radiologic images
•Videos allow for self-determined preparation	•Lecture duration appropriate	
•Videos are focused on learning objectives	•Possibility to ask questions	
•Short learning videos		
•Option of break-setting of videos is useful		
•Series of short videos appropriate		
**Suggestions for improvement**		
•Don’t read from the slides, speak freely	•Extend lecture duration	•Provide images to download
•Provide references to further information	•Waive buzz groups in favour of plenary discussion	•Provide images of high resolution (to zoom in)
•Provide scripts	•Less but longer buzz group discussions	•Provide explanation to the images in writing
•Put emphasis on exam preparation	•Smaller buzz groups	•Use cursor to explain images
•Provide objectives catalogue	•Provide solution to key questions to the entire group	•Clearly label pathological structures
	•Lecturers should take more time to correct students’ errors	•Provide comparative images of healthy subjects
		•Provide basics of clinical radiology (X-ray, CT, MRT)
		•Provide algorithm for radiological examination
		•Please be more reserved with radiologic specialties
		•Add lecture on duplex ultrasound

Lecturers stated that they would have liked more information on the level of students` knowledge to prepare lectures more targeted. Although ARS required continuous technical and administrative support, in the eyes of the lecturers, it stimulated the plenary debate on clinical cases. Buzz groups were difficult to track for lecturers because, for one thing, with VIC, students were prone to remain in silence, and secondly, there were too many groups to check on. In plenary, students showed great interest in clinical cases, however, with VIC, they were less well prepared than expected. Lecturers appreciated that with the VIC, emphasis was placed on consolidation of knowledge and practical application. However, they felt restricted in spontaneity and perceived themselves distanced and impersonal (Table3).

**Table 3:** Lecturer Survey After Completion of the Virtual Inverted Classroom in Clinical Radiology

	**Positive aspects**	**Negative aspects**
**Effort necessary for lecturer’s preparation**	•Like PIC	•Preparation of new learning videos needs initial additional effort •Prior knowledge of students not clear •A lot of slides are required
**Audience response system as tool for teaching**	•Stimulates debate on clinical case studies	•Sensitive technology
**Buzz groups**	•Only little guidance necessary •Works with different knowledge levels	•Frequently, students remain silent •Difficult to keep track •Support of individual buzz groups restricted by large number of groups
**Effect of the virtual inverted classroom on students**	•Great interest in clinical cases	•Students were inadequately prepared •Students can hide and disappear in the crowd
**Own perception of the role as lecturer**	•Consolidation and application rather than acquisition of knowledge •Seminar leader or tutor rather than a lecturer	•More a face than a person •Sometimes a voice from off-stage •Role as an entertainer •No commitment to the students •Option of spontaneity restricted
**Process and technical requirements**	•Efficient support and supervision	•Continued technical support necessary •Data network and hardware worthy of improvement
**General impression**	•Well organized •Practicable •Practice-oriented	•At that time without alternative •Technical problems •Ill-prepared students

## Discussion

We enforcedly implemented VIC in clinical radiology to prevent possible contagion during the COVID-19 pandemic. Our approach was based on our successful experience with PIC in the immediately preceding year [5] and on earlier publications on self-directed learning and the concept of inverted classroom [3, 4, 8, 17]. The study revealed that students’ overall impression of VIC was satisfactory, however, significantly poorer compared to the historical control of PIC. Deterioration can be accounted for by all aspects queried, but mainly by the buzz group, except for the audience response system. From the perspective of lecturers, criticism must be levelled at students’ preparation and active participation. However, in their final exam, students scored better with VIC than with PIC.

Due to the COVID-19 pandemic, we modified the existing concept of PIC of our clinical radiology lecture. Interactive and cooperative lecture elements had now to be conducted virtually to mitigate spread of infection. Although we attempted to conceive VIC as similar as possible to PIC, the two approaches simply were not the same: Before the start, because of the urgency, contrary to the PIC, we did not provide students with an introduction podcast. Instead, we left provision of orientation to the lecturers and thus, students were thrown in at the deep end, which could have resulted in uncertainty and restraint. It would be better to orient students on VIC and to make responsibilities including contribution to the discussion explicit from the outset [18]. However, we cannot rule out that social isolation during the COVID-19 pandemic led to emotional disruption which may have affected students’ perception. Another factor for a generally subdued note might have been the so called “Zoom fatigue” from virtual information overload during the pandemic [19]. A previous study shows that despite of advantages with online learning during the pandemic including flexibility, location-independence, and saving of time, students had experienced low motivation, concentration problems, difficulty to ask questions, loss of immediate feedback, lack of contact with peers, and family distractions [6].

With both VIC and PIC, students appreciated self-determined acquisition of knowledge, inter alia, through learning videos, because they could choose their own pace [2]. Anyway, they provided valuable suggestions for improvement which mainly concerned radiological images and explanations by lecturers. It might be beneficial if clearly labelled images and related information/articles could be shared through a central knowledge repository [20]. Although learning videos did not differ to those of PIC, students rated them somewhat worse, to which a generally deteriorated basic sentiment during the pandemic with increased expectations of clarity and conciseness could have contributed. After all, the pandemic gives reason and pressure to update internal learning resources [6] and to consider external online resources [21].

Smallest difference to PIC according to students’ assessment and best rating was achieved with the ARS which was used for processing KFQs during case-based discussions. According to lecturers, ARS stimulated the plenary debate. Overall, ARS use appears to be a suitable method to encourage individual engagement and to provide immediate relevant feedback in large groups, regardless of whether in-person or virtual.

Greatest difference to PIC and worst rating concerned the virtual buzz groups. Students complained about too large groups and challenging tasks, and lecturers bemoaned poor participation of students and poor assistance for student support. With PIC, buzz groups had been smaller and mostly permanent consisting of peers who sat next to each other. In turn, with VIC, large groups with randomly selected participants and social distance between group members might have caused loss of motivation and cooperation, once described as social floating. A previous study supports our findings and reported that students perceived leader-centred virtual activities such as pre-recorded presentations or traditional lecture-style teaching as equally effective, but participant-centred virtual activities such as virtual breakout room discussions with peers as less effective than face-to-face sessions [22]. Smaller and self-chosen groups as well as an appropriate number of assistant lecturers or student assistants, at least on-demand, for group support would be advantageous [18, 23]. Whether group process is more effective if KFQs and corresponding images are available for individual preparation already before the virtual classroom, could be evaluated in the future. Another method to facilitate group discussion is to provide thinking time before opening the breakout rooms [18].

Comments of both students and lecturers imply a considerable divergence in the idea of students’ current knowledge and skills to be built on. A solution might be to provide students with detailed information on pre-requisite knowledge and specify resources available from a central knowledge repository [20]. To avoid anonymity and facilitate collaboration, preparation for virtual lectures and/or voluntary lecture follow-ups could be organized in groups. In addition, positive mental-health related elements such as discussion rounds with peers could be encouraged and supported [6, 24, 25]. This might help students not only to keep social contacts but also to receive guidance through information overload to prevent burnout and anxiety [6].

For face-to-face conversation it is known that speakers adapt their messages to their listeners understanding. Both speakers and listeners collaboratively establish a common ground and then, iteratively refine utterances. Therefore, depending on emerging uncertainty of listeners, speakers tend to fragment their information. Although this strategy is cognitively demanding for the speaker, it avoids additional collaborative effort [26]. As uncertainty is mostly expressed non-verbally [18], with virtual conversation, the level of understanding is difficult to recognize and thus, conversational behaviour pattern might be disturbed. Whether immediate student feedback on ambiguities during the VIC can liven up the plenary discussion may be subject of future research. A previous study on distance teaching found a strong correlation between learning outcome and students rating of teachers response to questions and suggestions [9]. Debriefing of lecturers might disclose challenging situations and help to generate solutions. After all, faculty development training, networking, and mentoring, as well as data safety measures should be adapted to distance teaching [27].

Final grades, as the most objective performance indicators, went up with VIC. Although this is encouraging, we can only speculate about reasons. It might be that both, the concept of VIC and less distraction during exam preparation due to the pandemic have contributed to the good results.

Our study has some limitations. It was a single centre trial and only concerns the specialty of clinical radiology which may limit generalizability. Outcomes were compared with a historic control, that underwent PIC before the COVID-19 pandemic. Therefore, differences need to be interpreted with caution. As only 51% of students who underwent the VIC participated in the survey, probably those more satisfied or dissatisfied, the bias of self-selection could have affected outcomes. The outcomes of student and lecturer perception of VIC was based on subjective assessment and thus largely depends on individual evaluators. Finally, we only used summative assessment of learning success. An additional formative assessment of competences such as radiological reporting might have substantiated the outcomes.

## Conclusions

To conclude, clinical radiology VIC was a satisfactory alternative to PIC during the COVID-19 pandemic. However, our approach needs improvement concerning appropriate orientation of students before VIC and structure of buzz groups. Group size should not be too large and additional thinking time and individual support should be provided. Lecturers should receive immediate feedback on plenary ambiguities. Mental well-being of students and faculty participating in the VIC should be considered. Overall, an accordingly amended VIC might serve as a valuable complement to in-person lectures for the time after the pandemic to offer medical education independent of locations and to facilitate joint education in different universities and hospitals.

## Data Availability

The datasets generated and analysed during the current study are not publicly available due to requirements of the ethics committee but are available from the corresponding author on reasonable request.

## Abbreviations

ARS: Audience response system
KFQ: Key feature question
PIC: Physical inverted classroom
VIC: Virtual inverted classroom
